# Selectable Markers for Use in Genetic Manipulation of Extensively Drug-Resistant (XDR) *Acinetobacter baumannii* HUMC1

**DOI:** 10.1128/mSphere.00140-17

**Published:** 2017-04-26

**Authors:** Brian M. Luna, Amber Ulhaq, Jun Yan, Paul Pantapalangkoor, Travis B. Nielsen, Bryan W. Davies, Luis A. Actis, Brad Spellberg

**Affiliations:** a Department of Medicine, University of Southern California Keck School of Medicine, Los Angeles, California, USA; b Department of Molecular Microbiology and Immunology, University of Southern California Keck School of Medicine, Los Angeles, California, USA; c Department of Molecular Biosciences, University of Texas at Austin, Austin, Texas, USA; d Institute for Cellular and Molecular Biology, University of Texas at Austin, Austin, Texas, USA; e Department of Microbiology, Miami University, Oxford, Ohio, USA; Antimicrobial Development Specialists, LLC

**Keywords:** *Acinetobacter*, antibiotic resistance, genetics, Gram-negative bacteria, molecular biology

## Abstract

*Acinetobacter baumannii* is one of the most antibiotic-resistant pathogens in clinical medicine, and extensively drug-resistant (XDR) strains are commonly isolated from infected patients. Such XDR strains are already resistant to traditional selectable genetic markers, limiting the ability to conduct pathogenesis research by genetic disruption. Optimization of selectable markers is therefore critical for the advancement of fundamental molecular biology techniques to use in these strains. We screened 23 drugs that constitute a broad array of antibiotics spanning multiple drug classes against HUMC1, a highly virulent and XDR *A. baumannii* clinical blood and lung isolate. HUMC1 is resistant to all clinically useful antibiotics that are reported by the clinical microbiology laboratory, except for colistin. Ethical concerns about intentionally establishing pan-resistance, including to the last-line agent, colistin, in a clinical isolate made identification of other markers desirable. We screened additional antibiotics that are in clinical use and those that are useful only in a lab setting to identify selectable markers that were effective at selecting for transformants *in vitro*. We show that supraphysiological levels of tetracycline can overcome innate drug resistance displayed by this XDR strain. Last, we demonstrate that transformation of the *tetA* (tetracycline resistance) and *Sh ble* (zeocin resistance), but not *pac* (puromycin resistance), resistance cassettes allow for selection of drug-resistant transformants. These results make the genetic manipulation of XDR *A. baumannii* strains easily achieved.

**IMPORTANCE** Multidrug-resistant (MDR), extensively drug-resistant (XDR), and pan-drug-resistant (PDR) strains of *Acinetobacter baumannii* have frequently been characterized. The ability of *A. baumannii* to develop resistance to antibiotics is a key reason this organism has been difficult to study using genetic and molecular biology approaches. Here we report selectable markers that are not only useful but necessary for the selection of drug-resistant transformants in the setting of drug-resistant backgrounds. Use of these selectable markers can be applied to a variety of genetic and molecular techniques such as mutagenesis and transformation. These selectable markers will help promote genetic and molecular biology studies of otherwise onerous drug-resistant strains, while avoiding the generation of pathogenic organisms that are resistant to clinically relevant antibiotics.

## Abstract (precis)

Multidrug-resistant (MDR), extensively drug-resistant (XDR), and pan-drug-resistant (PDR) strains of *Acinetobacter baumannii* have frequently been characterized. The ability of *A. baumannii* to develop resistance to antibiotics is a key reason this organism has been difficult to study using genetic and molecular biology approaches. Here we report selectable markers that are not only useful but necessary for the selection of drug-resistant transformants in the setting of drug-resistant backgrounds. Use of these selectable markers can be applied to a variety of genetic and molecular techniques such as mutagenesis and transformation. These selectable markers will help promote genetic and molecular biology studies of otherwise onerous drug-resistant strains, while avoiding the generation of pathogenic organisms that are resistant to clinically relevant antibiotics.

## INTRODUCTION

Infections due to *Acinetobacter baumannii* have been identified by the Infectious Diseases Society of America (IDSA) and the Centers for Disease Control and Prevention (CDC) as a significant public health concern (1, 2). Of particular concern regarding *A. baumannii* is the exceptionally high frequency of extensively drug-resistant (XDR) strains (2–6). New prophylactic and therapeutic strategies are needed to combat such strains. The key to development of such novel approaches is a better understanding of pathogenesis of these infections (2, 4, 5, 7–9).

The prevailing dogma espouses that a fitness cost is always associated with the acquisition of antibiotic resistance (10–12). On the contrary, recent reports suggest that a fitness advantage exists for some specific antibiotic resistance mutations in *Salmonella enterica* serotype Typhi, *Pseudomonas aeruginosa*, and *A. baumannii* (13, 14). Thus, given the remarkable rise in frequency of XDR *A. baumannii* clinical strains, the use of an XDR strain is needed to best model clinically relevant infection dynamics in pathogenesis studies.

Unfortunately, our understanding of *A. baumannii* pathogenesis has been greatly hampered by a lack of available genetic manipulation techniques for highly resistant and clinically relevant strains (15–17). Advances in microbial genetics have provided tools such as transposon and site-directed mutagenesis that have rapidly improved our ability to study and manipulate organisms of interest (18–22).

However, such techniques require the use of a selectable marker to allow outgrowth of a desired mutant (23–27). Selectable markers take advantage of antibiotic resistance cassettes to allow for selection of mutants when grown under antibiotic selective pressure (28). The conundrum is that XDR *A. baumannii* strains are already resistant to commonly used selectable markers, precluding effective selection of such strains with most traditionally used selectable markers (17, 28–32). Thus, optimization of selectable markers is critical for the fundamental advancement of molecular biology research with XDR strains.

We have previously published that HUMC1, an XDR *A. baumannii* clinical blood and lung isolate resistant to all clinically reported antibiotics except colistin, is hypervirulent in murine models of infection (15, 16, 33, 34). Given its virulence and near-pan-drug-resistant status, intentional induction of colistin resistance in this strain, for example by inserting the MCR gene, would raise ethical concerns. Thus, while HUMC1 is a very useful model strain for studying pathogenesis, its intrinsic antibiotic resistance has made genetic manipulation challenging. To identify suitable selectable markers for such a resistant strain, we screened 23 compounds that constitute a broad array of antibiotics spanning multiple drug classes. Despite its intrinsic antibiotic resistance, we successfully identified selectable markers that are effective *in vitro* against HUMC1. Last, we show that supraphysiological levels of a drug, irrelevant to clinical use but achievable *in vitro* for selection of transformants, can overcome innate drug resistance displayed by an XDR strain.

## RESULTS

### MIC testing.

Based on results generated in the clinical microbiology laboratory at the hospital at which HUMC1 was isolated, *A. baumannii* HUMC1 was resistant to all clinical antibiotics except for colistin (Table 1). However, we noted that the tetracycline MIC of 12.5 µg/ml, while clinically defined as resistant due to an inability to achieve drug levels this high *in vivo*, was well within the range of concentrations achievable *in vitro* to enable selection of more-resistant clones. Furthermore, when we tested the related antibiotic doxycycline, we found a lower MIC (Table 1). Finally, two antibacterial agents that are not used clinically, puromycin and zeocin, also had activity against HUMC1 (Table 1).

**TABLE 1:** MIC results for drugs against *A. baumannii* HUMC1 and ATCC 17978^a^

Drug(s)	MIC(s) (µg/ml) of drug(s) against strain:		Method
	HUMC1	ATCC 17978	
Amikacin	>128	8	Vitek 2
Gentamicin	>128	8	Vitek 2
Aztreonam	64	16	Vitek 2
Ampicillin-sulbactam	16/8	1/0.5	Vitek 2
Piperacillin-tazobactam	>128/4	0.06/4	Vitek 2
Cefepime	32	2	Vitek 2
Meropenem	32	0.25	Vitek 2
Imipenem	16	0.25	Vitek 2
Ertapenem	128	4	Vitek 2
Doripenem	16	0.5	Vitek 2
Ciprofloxacin	>128	0.125	Vitek 2
Colistin	2	2	Vitek 2
Tigecycline	4	0.25	Vitek 2
Tellurite	62.5		Resazurin
Actinomycin D	>500	>500	Resazurin
Blasticidin S HCl	>2,500	>2,500	Resazurin
Doxycycline hydrochloride	0.25	<0.03125	Resazurin
Geneticin	>1,000	>1,000	Resazurin
Kanamycin	>50	>50	Resazurin
Puromycin	78.125	<39.06	Resazurin
Streptomycin	>50	>50	Resazurin
Tetracycline hydrochloride	12.5	0.125	Resazurin
Zeocin	12.5	6.25	Resazurin

a *A*. *baumannii* HUMC1 is sensitive to colistin, doxycycline, tetracycline (supraphysiological concentrations but attainable in vitro), puromycin, and zeocin.

### Tetracycline resistance.

Tetracycline resistance is conferred by the *tetA* gene from pBR322 and commonly found on many plasmids used for molecular biology. The fact that doxycycline retained activity against the strain despite tetracycline resistance suggested that the resistance observed was not due to the *tetA* gene. We confirmed that tetracycline resistance in the HUMC1 isolate was not due to the presence of the *tetA* gene. A BLAST search for *tetA* against the HUMC1 genome did not return any hits, and PCR for *tetA* using purified HUMC1 genomic DNA (gDNA) was negative as well. Colonies were successfully isolated by plating on agar plates supplemented with 50, 75, or 100 µg/ml of tetracycline, and no growth was observed for the nontransformed HUMC1 control, indicating the ability of the *tetA* gene to be used as a selectable marker in HUMC1, despite clinically defined tetracycline resistance.

**FIG 1:**
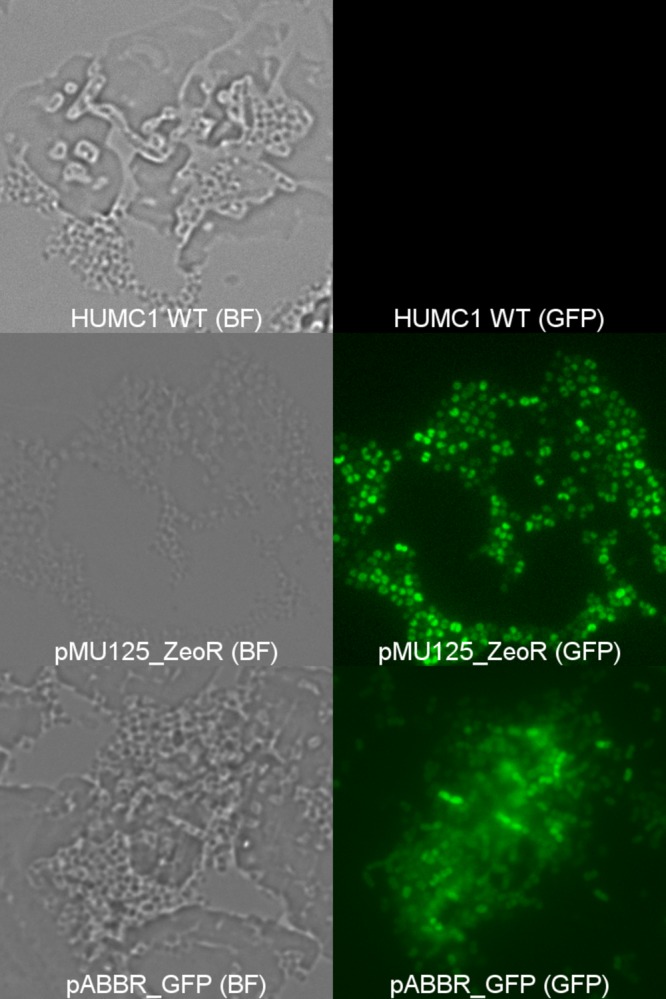
Successful transformation and expression of GFP in *A. baumannii* HUMC1 using plasmids containing zeocin (pMU125_GFP) or tetracycline (pABBR_GFP) resistance gene. The wild-type (WT) *A. baumannii* HUMC1 alone or carrying plasmid pMU125_GFP or pABBR_GFP is shown by bright-field microscopy (BF) or fluorescence microscopy (GFP). Magnification, ×1,000.

The purified pABBR_GFP plasmid was transformed into HUMC1 isolate, and transformants were selected by plating on tryptic soy agar (TSA) plate with 100 µg/ml of tetracycline. Expression of green fluorescent protein (GFP) was confirmed in transformed HUMC1 with nontransformed HUMC1 as a negative control using a fluorescence microscope (Fig. 1).

### Zeocin resistance.

**FIG 2:**
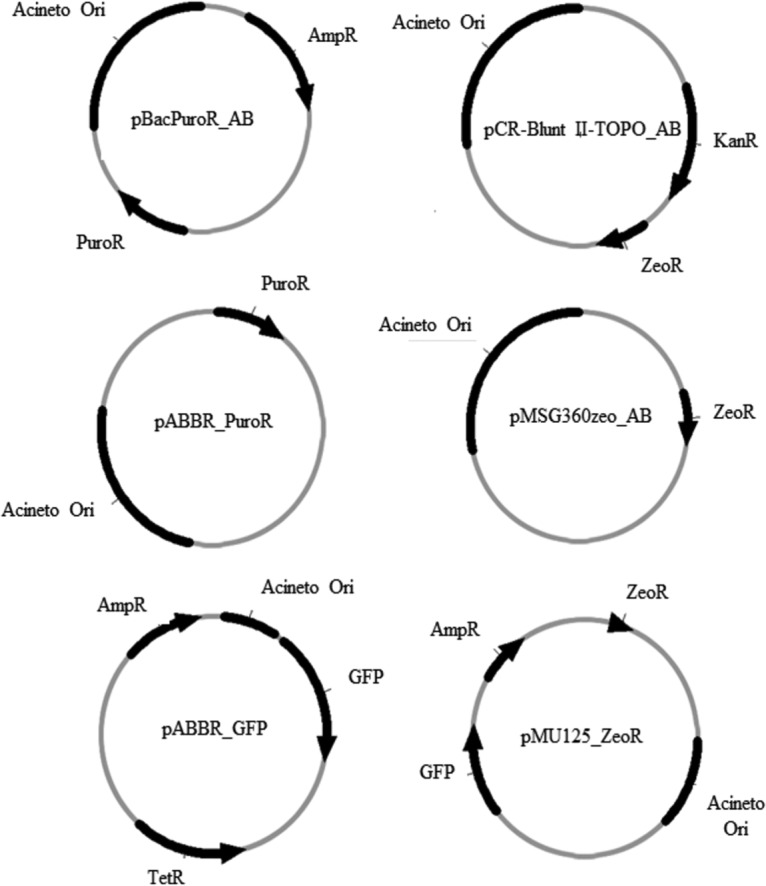
Plasmid constructs developed for this study. Constructs were developed by linearizing the vector backbone and insert by PCR, and assembly of the linear parts was performed by Gibson assembly.

Zeocin is an antibiotic that is not used clinically. Resistance to zeocin is conferred by the *Sh ble* gene. Unfortunately, plasmids that contain the *Sh ble* gene with an *Acinetobacter* origin of replication are not readily available, so we developed pMSG360Zeo_AB and pCR-Blunt II-TOPO_AB (Fig. 2). Successful transformants were selected by plating on low-salt Luria-Bertani broth (LB) agar supplemented with 250 µg/ml zeocin. The presence of the plasmid was further verified in the transformants by PCR.

In order to demonstrate efficacy of zeocin selection to maintain HUMC1 transformants, the *Sh ble* resistance gene from pCR-Blunt II-TOPO was cloned into pMU125 to form pMU125_ZeoR and was transformed into HUMC1 isolate. Transformants were selected for on low-salt LB agar supplemented with 250 µg/ml zeocin. Successful transformation of pMU125_ZeoR into HUMC1 was confirmed by fluorescence microscopy (Fig. 1).

### Puromycin resistance.

Resistance to puromycin is conferred by the *pac* gene encoding puromycin *N*-acetyltransferase (PAC). The *pac* open reading frame was cloned from pBacPuroR-NeoR by PCR and inserted into pABBR_MCS by the Gibson assembly method to form pABBR_PuroR (Table 2 and Fig. 2). The plasmid was sequenced, and it was confirmed that the *pac* open reading frame was in frame with the promoter and no mutations were present. The plasmid allowed for selection of puromycin-resistant colonies in *Escherichia coli*, but transformation of the plasmid was unable to confer puromycin resistance in *A. baumannii*.

**TABLE 2:** Description of plasmids used in this study

Plasmid	Resistance gene(s)^a^	Source and/or reference
pMol130-TelR	Tellurite	Addgene plasmid no. 50799 (30)
pBacPuroR-NeoR	Amp, neomycin, puromycin	Addgene plasmid no. 34921 (41)
pMSG360zeo	Zeocin	Addgene plasmid no. 27154 (42)
pCR-Blunt II-TOPO	Zeocin	ThermoFisher catalog no. K2800J10
pWH1266	Amp, Tet	43
pABBR_MCS	Amp, Tet	43
pBacPuroR_AB	Puro	This study
pABBR_PuroR	Puro	This study
pCR-Blunt II_AB	Zeocin	This study
pMSG360zeo_AB	Zeocin	This study
pABBR_GFP	Amp, Tet	This study
pMU125_ZeoR	Amp, zeocin	This study
pMU125	Amp	44

a Amp, ampicillin; Tet, tetracycline; Puro, puromycin.

We attempted a second plasmid construct design in which the *pac* resistance cassette from pBacPuroR-NeoR vector was left intact. pBacPuroR_AB was formed by cloning the *A. baumannii ori* region from pABBR_MCS and assembling it into a linearized pBacPuroR-NeoR using the Gibson assembly method (Table 2 and Fig. 2). Sanger sequencing was done to confirm the proper assembly of the construct. This second construct version allowed for selection of *E. coli* transformants, but once again we were unable to select *A. baumannii* transformants.

## DISCUSSION

Standard clinical definitions and classifications of drug sensitivity for microbes are based on achievable levels of antibiotics in the body (35). However, these definitions can be unnecessarily conservative when considering *in vitro* use as a selectable marker for genetic manipulation. It is possible to achieve significantly higher drug concentrations *in vitro* than *in vivo* (plasma, serum, bone, tissue, etc.). Here we have demonstrated that concentrations of tetracycline unachievable *in vivo* can be easily used *in vitro* for selection of “highly drug-resistant” mutants in a clinically drug-resistant strain. Furthermore, we found that the XDR strain was susceptible to several selectable markers that are not used as clinical antibiotics and also to a drug (doxycycline) that is used clinically but was not reported by the clinical microbiology laboratory. Thus, we emphasize the need to conduct systematic screens of potential selectable markers not limited by presumptions based on resistance profiles reported clinically.

Previous efforts have attempted to introduce and optimize standard genetic and molecular biology techniques in *A. baumannii* such as transformation, gene knockout, and transposon libraries (27, 32, 36). However, there are still relatively few molecular tools that have been validated for use in *A. baumannii* compared to other bacterial species such as *E. coli*, *Bacillus subtilis*, *Staphylococcus aureus*, and *Mycobacterium tuberculosis*. For example, there were no *Acinetobacter* plasmids available through Addgene.org (a nonprofit plasmid repository) (Cambridge, MA) at the time of this publication. Validation and standardization of these basic tools will benefit the research community in general and make *Acinetobacter* research more accessible.

We attempted to develop our constructs conferring resistance in one of two ways. First, the coding sequence (CDS) region of the antibiotic resistance gene (*tetA*, *Sh ble*, or *pac*) was cloned in frame with the *bla* promoter which is recognized by the highly conserved sigma-70 (*rpoD*) “constitutive housekeeping” promoter. The sigma-70 sigma factor is highly conserved in *E. coli* and *A. baumannii* so it was reasonable to hypothesize that *A. baumannii* transcription machinery would successfully recognize the *bla* promoter and express the transgene in a manner similar to that in *E. coli*. Sequencing of the assembled construct confirmed that the *pac* gene had replaced the *bla* open reading frame (ORF) in frame with the promoter. As that method did not work, we next tried to leave the promoter region of the *pac* gene intact and instead add the *A. baumannii ori* sequence to the pBacPuroR_NeoR plasmid. The promoter sequence differed from the *bla* promoter that was present in pABBR_MCS so it was reasonable that a change in the promoter sequence would improve gene expression; however, this approach was also unsuccessful.

Thus, we were unable to develop a functional puromycin selectable marker in *A. baumannii* despite the functional activity displayed by *E. coli* transformants. This difficulty could be due to the use of genetic elements that have not been optimized for expression in *A. baumannii* such as the promoter elements and codon sequence. While we were unable to express a functional *pac* gene in *Acinetobacter*, successful expression may be possible with a different promoter or codon optimized sequence. Additionally, the robustness of the antibiotic resistance conferred by the *Sh ble* and *tetA* genes used in the plasmids could be improved with similar promoter and codon optimization considerations.

We also observed that *A. baumannii* ATCC 17978 and HUMC1 were susceptible to drugs that are not used clinically, including puromycin and zeocin. This is most likely due to lack of exposure to these antimicrobial agents so selective pressure has not promoted mutants with resistance to these drugs. Recent publications have shown that other nonclinically relevant antimicrobials, such as tellurite, can be used for *in vitro* selection schemes (30, 37). Further effort to characterize selection systems, for drug resistance strains in particular, for basic science purposes continues to be of value.

A national surveillance study of U.S. intensive care units found that 50% of clinical isolates of *A. baumannii* were carbapenem-resistant, XDR strains (38). Further research is needed to better understand the basic physiology and host-pathogen interactions of the most difficult-to-treat and most lethal drug-resistant strains. Molecular tools such as selectable markers are needed to facilitate basic genetic studies and engender further research of these intractable strains. Our results enable transformation of antibiotic-resistant strains of *A. baumannii* by identifying alternative selectable markers and establishing effective constructs that are potentially useful in spite of an XDR phenotype.

## MATERIALS AND METHODS

### Bacterial strains.

*E. coli* DH5α, *A. baumannii* HUMC1 (15, 16, 33, 34), and *A. baumannii* ATCC 17978 were cultured using aseptic technique. Single colonies were first streaked out on tryptic soy agar (TSA) from frozen glycerol stocks. Single colonies were picked and used to inoculate overnight broth cultures in tryptic soy broth (TSB).

### Resazurin MIC assays.

The colometric resazurin assay was conducted as previously described (39, 40). Antibiotics were acquired from Sigma-Aldrich (St. Louis, MO) or ThermoFisher (Waltham, MA).

Overnight cultures of the bacteria (*A. baumannii* HUMC1 or ATCC 17978) grown in TSB were diluted 1:100 into Mueller-Hinton II (MH2) broth and subcultured in a shaking incubator at 200 rpm and 37°C until the optical density at 600 nm (OD_600_) reached 0.5. Bacteria were diluted to a working concentration of 1 × 10^6^ CFU/ml. The bacterial density was confirmed by plating serial dilutions on TSA and counting CFU.

MIC assays were conducted in standard, sterile, round-bottom (U-shaped), 96-well plates. Drug dilutions were done by serial twofold dilutions across plate columns. Wells of bacteria and media alone were included as positive and negative controls, respectively. One hundred microliters of 1 × 10^6^ CFU/ml bacterial culture was added to each one of the requisite wells. The plates were incubated for 24 h at 37°C. Twenty microliters of 0.1% resazurin dye was added to each well, and metabolism of the dye was measured after 1 h.

### Plasmids.

Details for the plasmids used in this study are listed in Table 2.

pMo130-TelR was a gift from Kim Lee Chua (Addgene plasmid no. 50799) (30). pBacPuroR-NeoR was a gift from Ben Lehner (Addgene plasmid no. 34921) (41). pMSG360zeo was a gift from Michael Glickman (Addgene plasmid no. 27154) (42).

### Primers.

Primers were purchased from Integrated DNA Technologies, Inc. (IDT) (Coralville, IA). Primer sequences are listed in Table 3.

**TABLE 3:** Primers used for this study

Plasmid or process and primer	Target	Template	Sequence^a^
pMSG360Zeo_AB			
ZeoF_pMSG_F	Linear pMSG360	pMSG360	CGTTCTTCTTCGTCATAACTTAATG
ZeoR_pMSG_R	Linear pMSG360	pMSG360	GAAACGCCTTAAACCGGAAAATTTTC
Zeo_OriF	*Acinetobacter ori*	pABBR_MCS	tttccggtttaaggcgtttcGGATTTTAACATTTTGCGTTG
Zeo_OriR	*Acinetobacter ori*	pABBR_MCS	agttatgacgaagaagaacgGATCGTAGAAATATCTATGATTATCTTG
pCR-Blunt II-TOPO_AB			
ZeoF_TOPO	Linear pCR-Blunt II-TOPO	pCR-Blunt II-TOPO	tcatagatatttctacgatcTTAAGGGCGAATTCTGCAG
ZeoR_TOPO	Linear pCR-Blunt II-TOPO	pCR-Blunt II-TOPO	aacgcaaaatgttaaaatccTCTATAGTGTCACCTAAATAGC
TOPOZeo_OriF	*Acinetobacter ori*	pABBR_MCS	GGATTTTAACATTTTGCGTTG
TOPOZeo_OriR	*Acinetobacter ori*	pABBR_MCS	GATCGTAGAAATATCTATGATTATCTTG
pMU125_ZeoR			
ZeoR_F	Zeocin resistance cassette	pCR-Blunt II-TOPO	agcgagtcagtgagcgaggaCGTTGGCTACCCGTGATATT
ZeoR_R	Zeocin resistance cassette	pCR-Blunt II-TOPO	ccgcatcaggcgctcttccgGATTAGCAGAGCGAGGTATGTAG
pABBR_GFP			
pABBR_GFP_F	*eGFP*	pMU125	agcgagtcagtgagcgaggaCCCTTTCGTCTTCAAGAATTCTC
pABBR_GFP_R	*eGFP*	pMU125	ccgcatcaggcgctcttccgTGAAGGCTCTCAAGGGCATC
pABBR_PuroR			
PuroF1	Linear pBacPuroR-NeoR	pBacPuroR-NeoR	GCGTCAGCGGGTGTTGGC
PuroR1	Linear pBacPuroR-NeoR	pBacPuroR-NeoR	CAGTCATAGCCGAATAGCCTCTCC
Puro_OriF1	*Acinetobacter ori*	pABBR_MCS	aggctattcggctatgactgGGATTTTAACATTTTGCGTTG
Puro_OriR1	*Acinetobacter ori*	pABBR_MCS	ccgccaacacccgctgacgcGATCGTAGAAATATCTATGATTATCTTG
pBacPuroR_AB			
PuroF2	Linear pBacPuroR-NeoR	pBacPuroR-NeoR	gaggtgccgccggcttccatTCAGGCACCGGGCTTGCGGGTCA
PuroR2	Linear pBacPuroR-NeoR	pBacPuroR-NeoR	aacgcagtcaggcaccgtgtATGACCGAGTACAAGCCCACGGTGC
Puro_OriF2	*Acinetobacter ori*	pABBR_MCS	ACACGGTGCCTGACTGCG
Puro_OriR2	*Acinetobacter ori*	pABBR_MCS	ATGGAAGCCGGCGGCACC
Confirmation PCR			
Zeo_Confir_F1	Zeocin resistance	pCR-Blunt II-TOPO	CGACGTGACCCTGTTCATC
Zeo_Confir_R1	Zeocin resistance	pCR-Blunt II-TOPO	TCGCCGATCTCGGTCAT
Zeo_Confir_F2	Kanamycin resistance	pCR-Blunt II-TOPO	CTTGTCGATCAGGATGATCTGG
Zeo_Confir_R2	Kanamycin resistance	pCR-Blunt II-TOPO	CTCTTCAGCAATATCACGGGTAG
Puro_Confir_F1	Puromycin resistance	pBacPuroR-NeoR	GTCACCGAGCTGCAAGAA
Puro_Confir_R1	Puromycin resistance	pBacPuroR-NeoR	GGCCTTCCATCTGTTGCT
Puro_Confir_F2	Amp resistance	pBacPuroR-NeoR	GCTATGTGGCGCGGTATTAT
Puro_Confir_R2	Amp resistance	pBacPuroR-NeoR	CTCCGATCGTTGTCAGAAGTAAG
TetR_ConfirF	Tetracycline resistance	HUMC1 genomic DNA	TAAATCGCCGTGACGATCAG
TetR_ConfirR	Tetracycline resistance	pAT04	GCGAGAAGCAGGCCATTAT

a Uppercase nucleotides represent exact matches to those in the template sequence. Lowercase nucleotides represent nucleotides in the 5′ adapter sequence needed for the Gibson assembly reaction but do not match the nucleotides in the template sequence.

### Transformation. (i) *Acinetobacter baumannii.*

*A*. *baumannii* cells were made electrocompetent according to published protocols (36). Briefly, 500 µl of an overnight culture was used to inoculate 50 ml of TSB medium, and the subculture was incubated until it reached an OD_600_ of 0.5. The cells were pelleted by centrifugation (10 min at 10,000 × *g*) and washed five times with 1 ml of 10% glycerol. The cells were separated into 100-µl aliquots and stored at −80°C for later use as we have previously described (33).

Plasmid DNA (25 ng) was mixed with electrocompetent cells, and the mixture was incubated on ice for 10 min. The mixture was transferred to a 1-mm cuvette and electroporated at 25 µF, 100 Ω, and 2.5 kV. Following electroporation, 900 µl of superoptimal broth with catabolite repression (SOC) was added to the cuvette, and the cells were transferred to a 2-ml microcentrifuge tube and then incubated in a shaking incubator at 200 rpm and 37°C for 1 h. The cells were then plated on TSA supplemented with 100 µg/ml tetracycline, 250 µg/ml zeocin, or 250 µg/ml puromycin.

### (ii) *Escherichia coli*.

Chemically competent or electrocompetent *E. coli* DH5α cells were used for the transformations. *E. coli* DH5α competent cells were made using the Mix & Go *E. coli* transformation kit per the manufacturer’s suggested protocol (catalog no. T3001; Zymo Research). Briefly, the DNA was incubated with competent cells on ice for 1 h prior to plating on TSA supplemented with 10 µg/ml tetracycline, 25 µg/ml zeocin, 125 µg/ml puromycin, 50 µg/ml kanamycin, or 100 µg/ml ampicillin. The concentration of antibiotics used for selection of *E. coli* was chosen according to the manufacturer’s directions. Electrocompetent *E. coli* DH5α cells were prepared using the same methods as described above for *A. baumannii.*

### Construct assembly.

The constructs were assembled using the Gibson assembly method (Fig. 2) (20). Overlap sequences for the vector and insert were determined using the NEBuilder assembly tool (New England BioLabs). Vector backbones were prepared by PCR amplification of plasmid DNA or by restriction enzyme digestion. Assembly of the parts to create the final constructs was accomplished using the NEBuilder HiFi assembly master mix per the manufacturer’s protocol. Briefly, the corresponding linearized vector (100 ng) and insert were added in a 1:2 molar ratio of vector to insert. The linear fragments were incubated with 10 µl of enzyme master mix at 50°C for 15 min. Two microliters of the assembly product was then used for bacterial transformation.

Preparation of the vector backbone and insert sequence for each plasmid were done as follows. For pBacPuroR_AB, the pBacPuroR-NeoR vector backbone was linearized by PCR, and the *A. baumannii ori* insert sequence was amplified by PCR from pABBR_MCS. For pABBR_PuroR, the pABBR vector backbone was linearized by PCR, and the puromycin resistance cassette insert sequence was amplified by PCR from pBacPuroR-NeoR. For pCR-Blunt II_AB, the pCR-Blunt II_AB vector backbone was linearized by PCR, and the *A. baumannii ori* insert sequence was amplified by PCR from pABBR_MCS. For pMSG360zeo_AB, the pMSG360zeo vector backbone was linearized by PCR, and *A. baumannii ori* insert sequence was amplified by PCR from pABBR_MCS. For pABBR_GFP, the linearized vector backbone was prepared by digestion with SapI, and the *gfp* insert sequence was amplified by PCR from pMU125. For pMU125_ZeoR, the linearized vector backbone was prepared by digestion with SapI, and the zeocin resistance cassette insert was amplified by PCR from pCR-Blunt II-TOPO.
